# Symptomatic Postoperative Discal Pseudocyst After Percutaneous Endoscopic Interlaminar Discectomy: Case Report and Literature Review

**DOI:** 10.1111/os.12863

**Published:** 2020-12-16

**Authors:** Wen‐bin Xu, Dan‐ju Wu, Chao Chen, Xing Zhao, Zhi‐jun Hu, Shun‐wu Fan, Xiang‐qian Fang

**Affiliations:** ^1^ Department of Orthopaedics, Sir Run Run Shaw Hospital Zhejiang University School of Medicine Hangzhou China; ^2^ Department of Pathology, Sir Run Run Shaw Hospital Zhejiang University School of Medicine Hangzhou China; ^3^ Department of Orthopaedics Yuhuan People's Hospital Taizhou China

**Keywords:** Percutaneous endoscopic interlaminar discectomy, Postoperative discal pseudocyst, Surgical treatment

## Abstract

**Background:**

A postoperative discal pseudocyst (PDP) is a cystic lesion that is formed in the operation area of the intervertebral disc, leading to recurrence or even worse symptoms. To our knowledge, to date, there is no research focusing specifically on PDP following percutaneous endoscopic interlaminar discectomy (PEID).

**Case presentation:**

We present the case of a 27‐year‐old man with L_5_S_1_ intervertebral disc herniation who was treated with PEID after failed conservative treatment. His leg pain was relieved immediately but reoccurred on the 40th day. MRI showed a PDP. Because loxoprofen and bedrest were ineffective and the patient was anxious, we performed a cystectomy. The patient's symptoms were significantly relieved, and a 6‐month follow up showed no recurrence both clinically and on MRI.

**Conclusion:**

A PDP is more likely to form using the interlaminar approach than the transforaminal approach. For patients with mental stress, severe pain, and neurological symptoms, surgery is suggested to remove the cyst. Discectomy cannot be performed when disc degeneration is mild.

## Graphical abstract

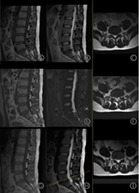

## Introduction

Lumbar disc herniation (LDH) is a common disease with an incidence of 1%–3%, usually manifested as low back pain radiating to the lower extremities, which seriously affects patients’ quality of life^1^. Surgical treatments for LDH include open discectomy (OD), microdiscectomy (MD), microendoscopic discectomy, percutaneous endoscopic lumbar discectomy (PELD), and lumbar interbody fusion. Among them, PELD has the lowest complication rate (5.8%)^2^ and has the advantages of shorter operation time, less paraspinal muscle damage, better bone structure preservation, and ideal maintenance of intervertebral disc height. There are two main approaches for PELD: the interlaminar approach and the transforaminal approach. Percutaneous endoscopic interlaminar discectomy (PEID) uses the posterior approach of the lamina, which is familiar to spinal surgeons. Patients with L_5_S_1_ disc herniation with high iliac crest or narrow intervertebral foramen and some patients with axillary type, migrated, calcified, or huge disc herniation could attain better decompression of discs with PEID.

However, there could be recurrence of PEID as in other nucleus pulposus resection surgeries. The re‐protruding mass may have other rare contents, such as postoperative discal pseudocysts (PDP). A PDP is a cystic lesion that forms in the operation area of the intervertebral disc. It compresses nerve roots and leads to recurrence of preoperative symptoms or even worse symptoms. Its morphology and imaging findings are similar to those of intervertebral disc cysts, both of which are cystic masses formed near the intervertebral disc, often containing bloody fluid. The difference between PDP and intervertebral disc cysts is that the cyst wall of PDP is incomplete, hence the name “pseudocyst.” Young *et al*. were the first to report two cases of PDP after MD surgery in 2009, which were referred to as postoperative annular pseudocysts at that time^3^. Kang *et al*. reported symptomatic PDP after PELD for the first time in 2011, with an incidence rate of 1.0% (15/1503), including 3 cases of PEID, with an incidence rate of 3.0% (9/298)^4^. Shiboi *et al*. retrospectively analyzed 359 patients after PELD and found only 1 symptomatic PDP with an incidence of 0.28%^5^. However, there is no research specifically on PDP after PEID. Moreover, the data on its clinical characteristics and pathogenesis are insufficient, and its treatment remains controversial.

We report one case of PDP after PEID and collected a total of four cases of PDP after PEID from the literature. The clinical characteristics were summarized, the possible mechanisms were discussed, and treatment experience was shared. This article aimed to strengthen the awareness of PDP after spinal surgery, to provide new ideas for PEID, and to identify recurrent root symptoms after surgery.

## Case Report

**Fig. 1:**
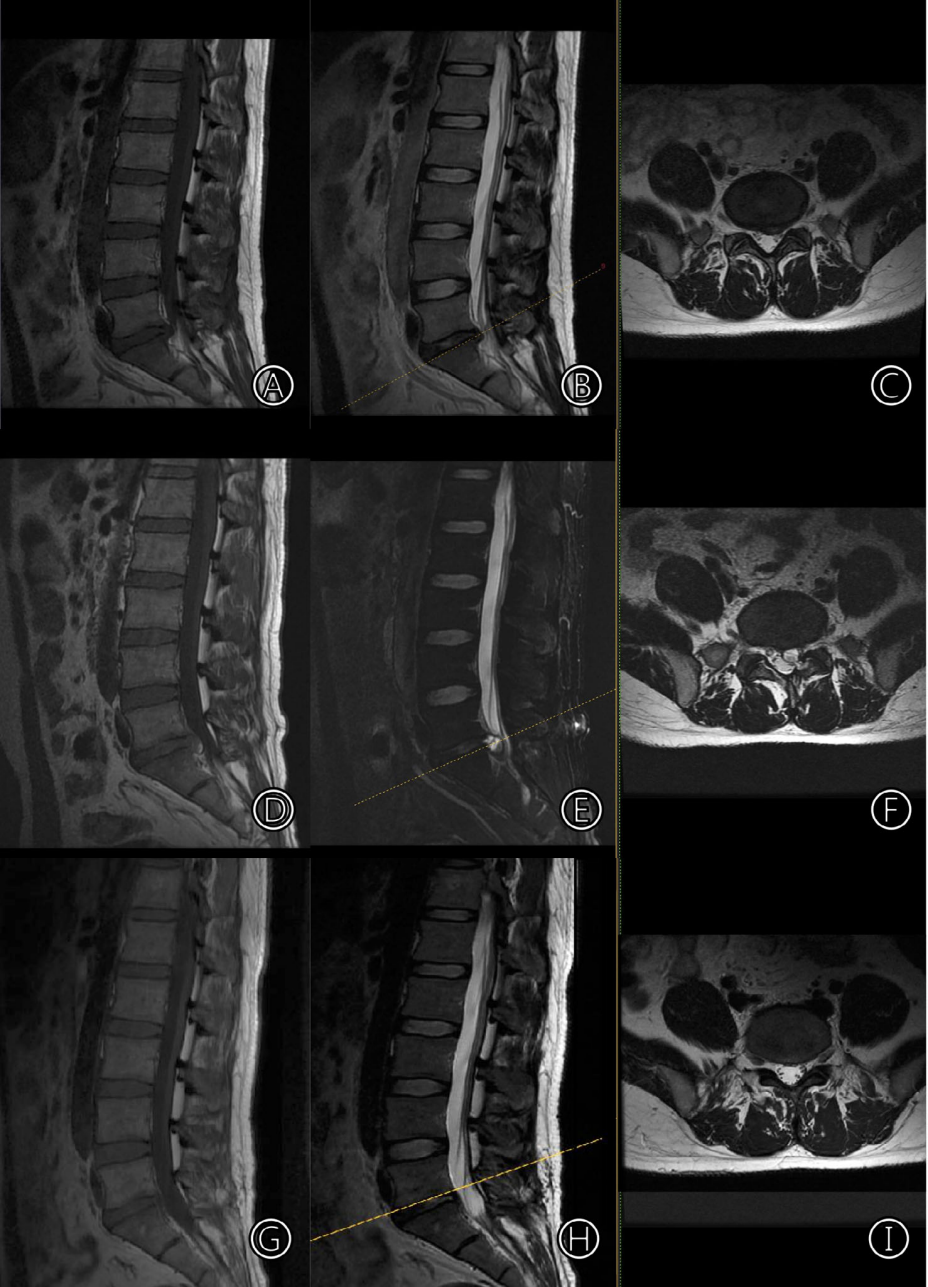
Preoperative MRI showed that the left part of the intervertebral disc of L_5_S_1_ protruded and compressed the nerve root of S_1_ (A–C). MRI on the 40th day after the first operation suggested a postoperative discal pseudocyst (PDP) (D–F). At the 6‐month follow‐up after the second operation, the MRI showed that the PDP had disappeared (G–I).

A 27‐year‐old man visited our hospital because of repeated left lower extremity radiation pain for 7 months. Physical examination showed normal sensation and muscle strength. The active straight raise test of the left leg was positive at 40°, and the enhancement test was also positive. MRI showed that the left part of the intervertebral disc of L_5_S_1_ protruded and compressed the nerve root of S_1_ (Fig. 1A–C). A 6‐month conservative treatment including bed rest and drugs was not effective. Furthermore, the patient's leg pain was aggravated for 1 month, with a visual analogue scale (VAS) score of 6 points. Therefore, we performed L_5_S_1_ PEID under general anesthesia. During the operation, axillary type L_5_S_1_ intervertebral disc herniation was confirmed, and nucleus pulposus resection, annuloplasty, and radiofrequency ablation were performed. After the operation, the patient's symptoms were significantly relieved, and the VAS leg pain score decreased to 1 point. On the second day after surgery, he could get up with waist protection and was discharged for follow‐up.

**Fig. 2:**
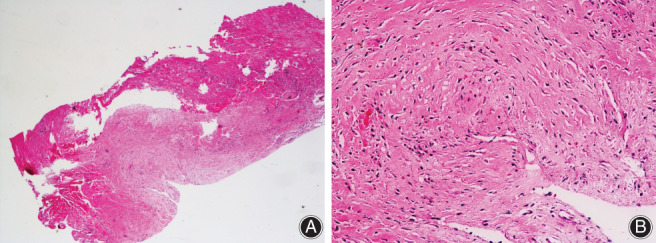
The pathology of the postoperative discal cyst showed fibrous tissue hyperplasia and local glassy changes (hematoxylin and eosin stain, A × 40, B × 200).

On the 40th day after the operation, the patient suddenly experienced radiating pain in the left lower extremity. The pain was more severe than that experienced before surgery, and he returned to our hospital again. Physical examination showed normal sensation and muscle strength. The active straight raise test of the left leg was positive at 20°, and the enhancement test was positive. MRI suggested PDP (Fig. 1D–F). Loxoprofen was administered for 1 week in addition to bed rest but was ineffective. The patient's VAS leg pain score was 7 points, and he was very anxious because of the recurring symptoms. We then performed a cystectomy under general anesthesia. During the operation, a pseudocyst was found in the former operation area of L_5_S_1_. The pseudocyst contained bloody fluid and high tension was evident, with the left S_1_ nerve root being tightly compressed. The histopathology of the cyst (determined using hematoxylin and eosin stain) showed fibrous tissue hyperplasia and local glassy changes (Fig. 2). After the operation, the patient's symptoms were significantly relieved, and the VAS leg pain score returned to 1 point. On the second day after surgery, the patient got up with waist protection. MRI was performed at the 6‐month follow up after surgery, and revealed that the PDP had disappeared (Fig. 1G–I). At the last follow‐up at 6 months, the patient's symptoms were fully relieved, and the VAS leg pain score was 0 points.

Four similar cases were found from the literature search and retained for further analysis (an overview is given in Table 1).

**TABLE 1:** PDP cases after PEID

							VAS			
Study	Year	Sex	Age (years)	Level	Duration (days)	Treatment	LBP	LBP	LP	LP
Kang *et al*.^4^	2011	Male	20	L_5_S_1_	11	PHL	5	1	7	1
	2011	Male	21	L_5_S_1_	32	PELD	5	0	4	0
	2011	Male	20	L_5_S_1_	31	PELD	N/A	N/A	N/A	N/A
Manabe *et al*.^6^	2019	Male	21	L_4–5_	42	PEID	N/A	N/A	N/A	N/A
Our case	2020	Male	27	L_5_S_1_	40	OD	2	1	7	0

LBP, lower back pain; LP, leg pain.; N/A, not available; OD, open discectomy; PDP, postoperative discal pseudocyst; PEID, percutaneous endoscopic interlaminar discectomy; PELD, percutaneous endoscopic lumbar discectomy; PHL, partial hemilaminectomy and discectomy; VAS, visual analogue scale.

## Discussion

### *Clinical Features*

Regarding the onset of PDP, Shiboi *et al*. reported a case of PDP after the transforaminal approach of PELD; the interval of recurring symptoms was 30 days^5^. The intervals of three PDP cases after the transforaminal approach of PELD reported by Chung were 33, 14, and 24 days, respectively^6^. In this study, the time interval of the five summarized cases of PDP after PEID averaged 31.2 ± 12.3 days. From this, we can conclude that the onset time of PDP after the interlaminar and transforaminal approaches of PELD is basically the same, both around 1 month after the first operation. Regarding the population and age of patients with PDP, the five cases of PDP after PEID were all in young men (21.8 ± 2.9 years), which is consistent with the published literature^7^. Furthermore, Aydin *et al*. reported PDP as more common among Asians^8^.

Wang *et al*. found that 93.6% of patients with PELD after surgery had a T1‐weighted medium or T2‐weighted high or medium signal mass on MRI 1 week after surgery, 82.8% of which disappeared 3 months after surgery^9^. However, the PDP discussed in this article are symptomatic masses. For this type of PDP, MRI is also the first choice for diagnosis, because it shows the nature of the mass and its relationship with the intervertebral disc. The main manifestation of PDP on MRI is an epidural cystic occupancy located on the ventral side. Similar to normal intervertebral discs, it displays a low signal on T1‐weighted imaging and a high signal on T2‐weighted imaging. In addition, the edge of the mass may show incomplete enhancement on the enhanced MRI. However, Aydin *et al*. reviewed the radiological data of 56 intervertebral disc cyst cases and pointed out that the signal intensity of the cysts was not regular. According to Aydin *et al*., 68.7% of cysts showed low signals on T1‐weighted images, 29% had equal signals, and 2% had high signals; on T2‐weighted images, 96% of cysts showed high signals, 2% had equal signals, and 2% had low signals; 92% of cases showed edge enhancement on enhanced MRI^9^. The MRI in our case was a T1 low signal and T2 high signal without enhanced MRI, in line with typical PDP. However, even patients with typical PDP findings on MRI cannot be diagnosed with PDP definitively because there is still the possibility of LDH recurrence^5^. According to the Pfirrmann grading system, the preoperative degeneration of the intervertebral disc was grade C in our case. It is speculated that preoperative mild disc degeneration (grades A to C) may be related to PDP, but this needs further study.

Discography can provide evidence of communication between the intervertebral disc and the cyst. Particularly when cysts cannot be found during surgery, intraoperative discography might be used to help locate those occult disc cysts and ensure that the cysts have been completely removed before the end of the operation^10^. Saydin *et al*. believe that discography is not necessary for the diagnosis of PDP, as MRI provides enough information^11^.

### *Particulars of the Interlaminar Approach*

Kang *et al*. retrospectively analyzed 1503 cases of LDH treated by PELD, including 1118 cases of L_4–5_ level through the transforaminal approach, among which 6 cases of PDP occurred, with an incidence rate of 0.54%; 330 cases were of L_5_S_1_ level through the interlaminar approach by PEID, among which 9 cases of PDP occurred with an incidence rate of 2.73%. Therefore, Kang suggested that the interlaminar approach was more likely to form PDP than the transforaminal approach^4^. We summarized a total of 190 patients with PELD who underwent surgery by the same surgeon in our hospital from January 2017 to December 2019, including 165 cases using the transforaminal approach; among them, 1 PDP occurred, and the incidence rate was 0.6%. The interlaminar approach was used for 25 cases, among which 1 PDP occurred, with an incidence rate of 4.0%, supporting Kang's conclusion. The possible reasons for PDP after PELD include the use of saline during surgery, which results in residual fluid in the operation area, electrocoagulation, vaporization, and other steps that cause inflammation reaction. Furthermore, the interlaminar approach involves separating the ligamentum flavum, the dural sac, and other structures to expose the posterior part of the herniated disc. It also involves exposing and electrocoagulating a larger range of the posterior longitudinal ligament, which exacerbates the inflammation in this ligament, the annulus fibrosus, and surrounding tissues, leading to intraspinal adhesion. These factors could explain the higher incidence of PDP through the interlaminar approach than that the transforaminal approach.

### *Pathogenesis*

Histologically, the wall of PDP mainly consists of dense fibrous connective tissue without epithelial lining, with serous or mucinous fluid inside^8^. The exact pathogenesis of PDP is not yet clear, but there are three major hypotheses in the literature: response to epidural hematoma; pseudomembrane formation after local annulus fibrosus tear and disc degeneration; and inflammatory response to protruding nucleus pulposus. The epidural hematoma hypothesis states that epidural venous plexus hemorrhage followed by reactive inflammation leads to cyst formation. Hemosiderin deposits found in the cyst wall support this hypothesis^12^. However, if the cyst is the result of a epidural blood vessel rupture rather than an annulus fibrosus tear, there should be no communication between the disc and the cyst. According to the reactive pseudomembrane theory proposed by Kono *et al*., the local disc degenerates and fluid leaks into the epidural space, causing an inflammatory reaction and, finally, the formation of a pseudomembrane^13^. Chung *et al*. assumed that the axial load pumps the liquid and blood of the mildly degenerated intervertebral disc through the annulus fibrosus fissure to the posterior space, resulting in a pseudocyst^6^. Histological fibrous connective tissue, imaging of the annulus fibrosus fissure, and communication between the disc and the cyst supports this hypothesis. In our case, the pathology of the cyst showed fibrous tissue hyperplasia and local glassy changes, which supports the third hypothesis by Chung.

### *Treatment*

#### *Conservative or Surgical Treatment*

There are different opinions about the treatment of PDP in the literature. Demaerel *et al*. reported the first case of a spontaneous L_1–2_ intervertebral disc cyst successfully treated conservatively. The symptoms were relieved after 3 weeks, and the cyst was determined to be reabsorbed on 4‐month MRI. Takeshima *et al*. reported a case of a spontaneous L_3–4_ intervertebral disc cyst. The symptoms resolved, and the cyst disappeared after 5 months of medication^12^. Both cases had no history of surgery and were intervertebral disc cysts rather than PDP. Chung reported 12 cases of PDP after nucleus pulposus resection (9 MD and 3 PELD), 6 of which were treated conservatively; on average, the cysts were reabsorbed 82.7 days later^4^. In conclusion, for patients with less severe pain and no neurological symptoms, conservative treatment can be considered, including bed rest, non‐steroid anti‐inflammatory medication, and physical therapy.

Kang *et al*. analyzed 15 cases of PDP after PELD and concluded that there was no significant difference between surgery and conservative treatment for PDP^1^. However, 1 of the patients still had a PDP progressing at 9 months. In the disc cyst cases reviewed by Aydin *et al*., three resolved spontaneously after conservative treatment, while five patients underwent surgery after conservative treatment failure^14^. Among the 126 disc cysts cases summarized by Park, only 19 patients (15%) received conservative treatment; their results were inconsistent and some did not improve^13^. Thus, conservative treatment takes longer and has unpredictable results. In addition, recurrent symptoms often have a great psychological impact on postoperative patients. Long‐term medication may lead to liver and kidney function damage, and long‐term bed rest may cause muscle atrophy. Therefore, we believe that for patients with severe symptoms, surgery can provide more timely and complete relief of pain and neurological symptoms. It is also suggested for cases in which the diagnosis of the mass cannot be determined.

### *Surgical Techniques*

CT‐guided percutaneous aspiration for the treatment of lumbar intervertebral disc cysts achieved complete relief in 88% of patients. There was no significant difference in the treatment effect of cyst aspiration with or without steroid injection^15^. However, the recurrence rate of aspiration is high, and it is not recommended as the first choice^16^. Manabe *et al*. treated PDP after PEID by injecting 2 mL of 1% lidocaine and dexamethasone into a cyst. The symptoms were alleviated, but recurred 2 weeks later, and PDP was finally removed by a second PEID^14^. In addition to the classic open surgery, PELD is also a surgical method for intervertebral disc cysts, which not only enables histopathology but also quick recovery with a low recurrence rate^17^. During PELD, if a pseudomembrane exists, hemostasis and smooth drainage are suggested.

Whether discectomy is needed during the second operation is controversial. Some authors have pointed out that if there is obvious communication between the corresponding intervertebral disc and cyst, the cyst and the corresponding intervertebral disc should be removed at the same time. Aydin *et al*. supported the simultaneous removal of the cyst and intervertebral disc. They believed that more radical resection can reduce the risk of recurrence^8^. Arslan *et al*. (2014) also performed a discectomy^16^. Park *et al*. compared 126 patients with intervertebral disc cysts and suggested that it was safe and effective to remove the cyst only, and no recurrence was found^18^. In Park *et al*., the degeneration of the intervertebral disc was relatively mild. Moreover, because the patients are mostly young, discectomy might affect their biomechanics and lead to spinal instability. In our case, we chose the classic open cyst resection but discectomy was not performed. At 6‐month follow up, the effect was satisfactory and there were no recurring symptoms.

## Conclusion

A PDP is more likely to form using the interlaminar approach (PEID) than the transforaminal approach. For patients with mental stress, severe pain, and neurological symptoms, surgery is suggested to remove the cyst. Discectomy cannot be performed simultaneously when disc degeneration is mild.
